# Prescribing Pattern of Antiseizure Drugs in Pediatric Epilepsy Patients at a Tertiary Care Hospital in New Delhi: A Cross-Sectional Study

**DOI:** 10.7759/cureus.110185

**Published:** 2026-06-03

**Authors:** Divya Chamaria, Kamlesh Garg, Rachna Sehgal, Hitesh Verma, Lalit K Gupta

**Affiliations:** 1 Pharmacology, Lady Hardinge Medical College, New Delhi, IND; 2 Pharmacology, Vardhman Mahavir Medical College and Safdarjung Hospital, New Delhi, IND; 3 Pediatrics, Vardhman Mahavir Medical College and Safdarjung Hospital, New Delhi, IND; 4 Anatomy, Lady Hardinge Medical College, New Delhi, IND

**Keywords:** antiseizure drug, pediatric epilepsy, polytherapy, prescription pattern, valproate

## Abstract

Background: Epilepsy is a disorder of the brain characterized by recurrent seizure activity. The aim of the present study was to analyze the pattern of antiseizure drug prescriptions in pediatric patients with epilepsy.

Methodology: An observational cross-sectional study was conducted on 196 patients with epilepsy. The primary objective was to describe the prescription pattern of antiseizure drugs in pediatric patients suffering from epilepsy using World Health Organization prescribing indicators. Data on the prescribed antiseizure drugs, such as name (generic or branded), frequency, monotherapy, and polytherapy, were also recorded.

Results: Among the 196 participants, the largest proportion (77; 39.29%) belonged to the 10-12 year age group. Male patients (137; 69.9%) predominated over female patients (59; 30.1%). Generalized tonic-clonic seizures (81; 41.33%) were the most common type of epilepsy, followed by epileptic spasms (57; 29%). Polytherapy was observed in 128 (65.30%) patients, while dual therapy and monotherapy were seen in 54 (27.55%) and 14 (7.14%) of patients, respectively. Valproate was the most prescribed antiseizure drug (122; 62.76%), followed by phenytoin (38; 19.3%) and levetiracetam (16; 8.16%). The average number of drugs per prescription was three. Generic prescribing accounted for 123 (63%) of prescriptions and 162(82.66%) of drugs were prescribed from the essential drug list. No injectable drugs were prescribed.

Conclusion: Valproate was the most prescribed antiseizure medication in pediatric epilepsy patients. Polytherapy was frequently observed. Most drugs were prescribed in generic form and from the essential medicines list.

## Introduction

Epilepsy is a neurological disorder characterized by the occurrence of recurrent epileptic seizures. An epileptic seizure represents a clinical manifestation resulting from abnormal, excessive, and synchronous electrical discharges from neurons in the brain [1]. These events may present as sudden and transient episodes arising from abnormal neuronal activity within the central nervous system. The severity and clinical presentation can vary widely, ranging from subtle manifestations that may not be easily recognized by observers to more obvious convulsive seizures [2].

Epilepsy is a common neurological condition in the pediatric population, with approximately 6-7% of children affected. According to the World Health Organization (WHO), nearly 50 million people worldwide live with epilepsy, and about 80% of these individuals reside in developing countries [3].The global prevalence of active epilepsy is estimated to be around 4-5 cases per 1,000 population. In India, the prevalence rate ranges from approximately 4.15 to 7.03 per 1,000 individuals [4]. Among newly diagnosed cases, about 60% are focal (partial) seizures, while the remaining 40% are generalized seizures.

Epilepsy is frequently associated with several comorbid conditions that can significantly affect the prognosis and quality of life of affected individuals. Although increasing attention is being given to these comorbidities, limited information is available regarding optimal strategies for their screening and the influence of treatment on long-term outcomes.

The primary approach to epilepsy management involves the use of antiseizure medications. These drugs are broadly categorized into older and newer antiepileptic agents. Conventional drugs include valproic acid, phenytoin, carbamazepine, phenobarbital, and primidone, whereas newer agents include lamotrigine, topiramate, levetiracetam, oxcarbazepine, and gabapentin. In addition, benzodiazepines such as midazolam, clonazepam, diazepam, and clobazam are frequently used as adjunctive therapy in seizure management [5]. The present study aimed to evaluate the prescribing patterns of antiseizure medications in pediatric patients with epilepsy using the WHO prescribing indicators [6].

## Materials and methods

Study design and setting

The research was conducted on 196 pediatric epileptic patients in the Department of Pharmacology, in collaboration with the Department of Pediatrics at Vardhman Mahavir Medical College and Safdarjung Hospital, New Delhi, 110029. In this descriptive observational cross-sectional study, the total duration was 18 months (July 2022 to January 2024). The study was conducted after obtaining ethical clearance. The study was conducted as per the guidelines of Indian Good Clinical Practice (GCP), as harmonized with International Council for Harmonization of Technical Requirements for Pharmaceuticals for Human Use - Good Clinical Practice ICH-GCP guidelines [7]. Written informed consent from parents/caregivers of patients aged 1-12 years was obtained from all enrolled patients after informing them completely about the study.

Eligibility criteria

Pediatric patients aged 1-12 years of both genders suffering from epilepsy who have been prescribed antiseizure drugs for at least one month were included in the study. Pediatric patients with non-seizure indications of antiseizure drugs, such as pain, migraines, and mood disorders, were excluded from the study.

Sample size and sampling technique

The estimated sample size was calculated using the formula: n= \frac{z^{2}p(1-p)}{d^{2}}

with confidence interval (Z) = 1.96, prevalence (P) of epilepsy in the pediatric age group = 0.5, and precision (d) = 0.05 [8]. The estimated sample size (n) was 384. Since our study population is limited by size, we take the finite population (T) as 400 and the adjusted sample size(s) was 196 obtained using the formula: s= \frac{n}{1+\frac{(n-1)}{T})}

Study parameters

The study was conducted in the department after obtaining written informed consent from the patients’ parents or caregivers. Data were collected using a pre-structured case record form (CRF). The required information included patient sociodemographic details (age, gender, locality) and type of epilepsy. The prescription pattern of antiseizure drugs was analyzed by using WHO prescription indicators. There are five WHO prescriptions indicators: (i) Average number of drugs per encounter C=B/A, (ii) Average number of antiseizure drug per encounter G = (F/A) × 100%, (iii) Percentage of drugs prescribed by generic name (E) = (D/B)×100%, (iv) Percentage of encounters with an injection prescribed I = (H/A) × 100%, and (v) Percentage of drugs prescribing from essential drug list or formulary K = (J/B) × 100%, where A= total number of encounters, B= total number of drugs prescribed during these encounters, D= total number of generic drugs prescribed, F= total number of patients who received antiseizure drugs, H= total number of patients who received one or more injections, and J= total number of essential drug list (EDL) drugs prescribed. The average number of antibiotics is also one of the WHO prescribing indicators.

Data collection and management

Data were obtained from the outpatient department during patient visits to reduce recall bias and to ensure accurate and complete information. The collected data were then entered into Microsoft Excel (Microsoft Corporation, Redmond, USA) using anonymized patient identification codes to protect confidentiality. To ensure reliability, a randomly selected portion of the data was cross-checked with the original records for verification.

Statistical methods

The collected data were analyzed using Stata 17.0 (Stata Corp LLC, Texas, USA). Descriptive statistical methods were used for data analysis. Simple tables and cross-tabulations were generated to summarize the study findings. Continuous variables, such as the number of drugs prescribed per encounter, were expressed as mean ± standard deviation, while categorical variables, including sex distribution and diagnosis, were presented as frequencies and percentages. Wherever necessary, subgroup analyses were performed to further interpret the data. The results were also illustrated using appropriate graphs and charts to enhance clarity and interpretation of the findings.

Ethics approval

Before initiation, the study protocol was reviewed and approved by the Institutional Ethics Committee of Vardhman Mahavir Medical College and Safdarjung Hospital (No. IEC/VMMC/SJH/Thesis/06/2022/CC-239). Written informed consent was obtained from all participants before data collection commenced.

## Results

The mean age of the study population was 7.10 ± 3.63 years. Seventy-seven (40%) study participants were 10-12 years of age. There was a high proportion of male patients (137; 69.90%) compared to female patients (59; 30.10%). The majority of prescription encounters were generalized tonic-clonic seizure (GTCS) (81; 41.33%) and epileptic spasm (57; 29.08%), followed by focal seizure (32; 16.33%). Monotherapy was observed in 14 (7.14%) of prescriptions encountered in the study. Dual therapy was observed in 54 participants (28%), and the remaining were on polytherapy (128; 65%). Valproate was the most prescribed antiseizure, comprising 122 (63%) of the total prescriptions, followed by phenytoin 38(20%) and levetiracetam 16(8%). The most prescribed concomitant medication was calcium, followed by vitamin supplements. 

Table 1 presents the sociodemographic characteristics of the 196 participants. Most participants belonged to the 10-12-year age group (77; 39.29%), followed by 4-6 years (46; 23.47%), 1-3 years (45; 22.96%), and 7-9 years (28; 14.29%). The mean age of the study population was 7.10 ± 3.63 years. Boys constituted a higher proportion of the participants (137; 69.90%) than girls (59; 30.10%). Only three participants (1.53%) had a history of pre-existing drug allergy, while the majority (193; 98.47%) reported no such history.

**Table 1:** Baseline sociodemographic and clinical characteristics of study participants (n = 196)

Variable	Category	Number (n)	Percentage (%)
Age group (years)	1–3	45	22.96
	4–6	46	23.47
	7–9	28	14.29
	10–12	77	39.29
	Mean ± SD	7.10 ± 3.63 years	—
	Total	196	100.00
Gender	Boys	137	69.90
	Girls	59	30.10
	Total	196	100.00
Pre-existing allergy	Yes	3	1.53
	No	193	98.47
	Total	196	100.00

Table 2 depicts the distribution of seizure types among pediatric patients according to the International League Against Epilepsy (ILAE) 2022 seizure classification [9] (n = 196). Generalized onset seizures were the most common, accounting for 81 (41.33%) of cases, followed by epileptic spasms (57; 29.08%). Focal onset seizures were observed in (32; 16.33%) of patients. Other generalized seizure types such as myoclonic, tonic, and clonic seizures were less frequent. Acute symptomatic seizures in the form of febrile seizures were reported in 12 (6.12%) of the study population.

**Table 2:** Distribution of seizure types according to ILAE 2022 classification (n = 196) ILAE: International League Against Epilepsy

ILAE Classification	Seizure Type	Number of Patients	Percentage (%)
Focal onset seizures	Focal seizure	32	16.33
Generalized onset seizures	Generalized tonic–clonic seizure	81	41.33
	Epileptic spasms	57	29.08
	Myoclonic seizure	7	3.57
	Tonic seizure	4	2.04
	Clonic seizure	3	1.53
Acute symptomatic seizures	Febrile seizure	12	6.12
Total		196	100

Table 3 depicts the core prescribing indicators proposed by the WHO to evaluate the rational use of medicines in healthcare settings. The average number of drugs per prescription was 3, which was higher than the WHO-recommended range (1.6-1.8). Generic prescribing was observed in 123 (63%) of prescriptions, while 162 (82.66%) of the drugs were prescribed from the essential drug list, showing a high incidence of rational prescribing. No injectable or antibiotic drugs were prescribed in the studied prescriptions.

**Table 3:** Prescription pattern of antiseizure drugs according to WHO core prescribing indicators (n = 196 prescriptions)

WHO Prescribing Indicator	Observed Value in the Study	WHO Standard/Reference Value	Interpretation
Average number of drugs per prescription	3	1.6–1.8	Higher than recommended
Average number of antiseizure drugs per prescription	2	Not specifically defined	Depends on the disease condition
Average number of antibiotic drugs per prescription	0	As low as possible	Not applicable
Percentage of drugs prescribed by generic name	63%	100%	Lower than recommended
Percentage of encounters with injectable drugs	0%	13–24%	Acceptable/rational
Percentage of drugs prescribed from the essential drug list (EDL)	82.66%	100%	Lower than recommended

**Figure 1:**
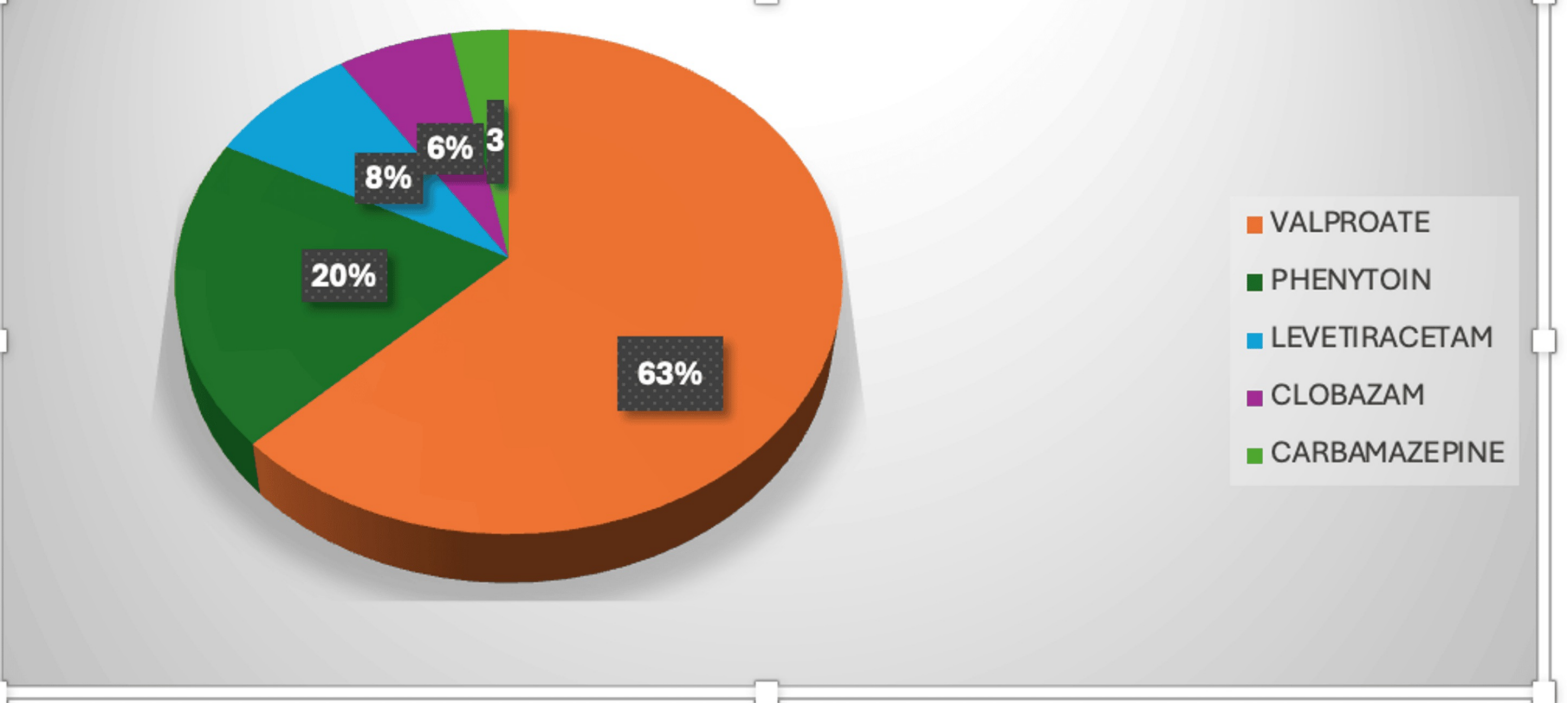
Distribution of antiseizure drugs prescribed among pediatric epileptic patients

Figure 1 summarizes the distribution of first-line antiseizure drugs prescribed among pediatric epilepsy patients. Valproate was the most frequently prescribed first antiseizure drug, accounting for 122 (62.76%) of prescriptions, followed by phenytoin (38; 19.90%). Other commonly used drugs included levetiracetam (16; 8.16%), clobazam (12; 6.12%), and carbamazepine (6; 3.06%). The predominance of valproate indicates its wide utilization as an initial therapy for seizure management in the studied pediatric population.

Table 4 presents the duration of antiseizure drug prescription among pediatric epilepsy patients (n = 196). The duration of antiseizure drug prescription among the study participants varied from one to three months. Many patients (78; 39.80%) received prescriptions for a duration of three months, followed by two months (63; 32.14%) and one month (55; 28.06%). This indicates that most patients were provided medications for longer follow-up intervals.

**Table 4:** Duration of antiseizure drug prescription among pediatric epilepsy patients (n = 196)

Months of prescribed treatment	Freq. of participants	Percent (%)
1	55	28.06
2	63	32.14
3	78	39.80
Total	196	100.00

**Figure 2:**
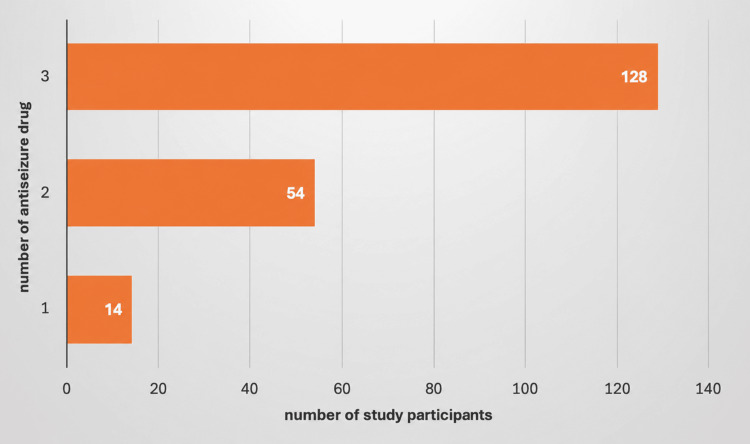
Distribution of the number of antiseizure drugs prescribed among pediatric epileptic patients

Figure 2 illustrates the distribution of the number of antiseizure drugs prescribed among pediatric epilepsy patients. The analysis of prescriptions revealed that most patients (128; 65.31%) were prescribed three antiseizure drugs, indicating the predominance of polytherapy in the study population. Dual therapy was observed in 54 (27.55%) patients, while only 14 (7.14%) patients received monotherapy.

## Discussion

Epilepsy is one of the most prevalent neurological disorders worldwide, and its burden continues to increase. In the present study, the highest proportion of patients belonged to the 10-12-year age group, comprising 77 participants (39.29%) of the total study population. These findings differ from those reported by Khoshdel et al., who observed that most patients receiving antiseizure medications were in the 2-6-year age group [10].

In our study, male patients were the most to receive antiseizure therapy (137; 69.9%), while female patients accounted for 59 (30.1%). A similar gender distribution was observed in a study conducted by Vijayarangan et al., where 55% of participants were male, and 45% were female [11]. This male predominance is consistent with previous studies that also reported a greater frequency of epilepsy among male patients. For example, Joshi et al. reported that 65.7% of epilepsy patients were males in their study population [12].Similarly, Kaur et al. documented 55% male and 45% female participants among pediatric epilepsy patients [13].The higher prevalence among male patients may be attributed to biological, genetic, and healthcare-seeking factors.

Regarding seizure type, the most frequently observed condition in our study was GTCS, representing 81 (41.3%) of cases, followed by epileptic spasm (57; 29%). Comparable findings were reported by Lakshmi et al., who documented generalized epilepsy in approximately 20% of study participants [14].Kaur et al. observed that generalized epilepsy accounted for 78% of pediatric cases [13]. Joshi et al. also reported a slightly higher prevalence of generalized seizures (53%) compared to focal seizures (47%) [12]. The predominance of generalized seizures in pediatric populations may be related to developmental neurophysiological factors and the higher occurrence of syndromes such as West syndrome in early childhood.

Most patients in our study were treated with combination therapy. More than two antiseizure medications were prescribed in combination in 128 (65%) of the study population. Monotherapy was prescribed in only 14 (7.10%) cases, whereas dual therapy, most commonly the combination of valproate and levetiracetam, was observed in 54 (27.55%) participants. Polytherapy may sometimes provide better seizure control in patients with difficult-to-manage epilepsy. However, this finding contrasts with the study conducted by Dave et al., where 71% of patients were managed with monotherapy, and the most frequently prescribed drug combination was carbamazepine with clobazam (7%) [15].This finding contrasts with several earlier studies that reported a higher prevalence of monotherapy in epilepsy management. Kaur et al. reported that 92% of pediatric patients were treated with monotherapy, while only 8% received polytherapy [13]. Similarly, Kalinina et al. reported that 61.7% of epilepsy patients remained on monotherapy, whereas only 18.5% were on polytherapy [16]. However, the higher use of polytherapy in the present study may be attributed to the tertiary-care hospital setting, where more complex or refractory epilepsy cases are frequently managed. Many patients attending our tertiary-care center were on long-term follow-up with recurrent seizures, prior failure of monotherapy, and referrals from nearby smaller hospitals and clinics for further management. These clinical circumstances may possibly explain the higher use of polytherapy observed in our study population.

Among all antiseizure medications, valproate was the most prescribed antiseizure medication, comprising 122 (62.76%) of the total prescriptions, followed by phenytoin (38; 19.3%) and levetiracetam (16; 8.16%) in first-line therapy. In contradiction to this, in another study done by Dave et al., the most commonly prescribed antiseizure medication was carbamazepine (41%), with sodium valproate being the next most used (38%) [15]. These findings are consistent with earlier reports demonstrating the widespread use of valproate in epilepsy management due to its broad-spectrum efficacy. Joshi et al. reported sodium valproate as the most commonly prescribed antiepileptic drug (49.6%), followed by clobazam, levetiracetam, carbamazepine, and phenytoin [12]. Similarly, Kaur et al. observed that valproate was the most frequently prescribed drug in monotherapy (44.56%) [13]. The high utilization of valproate may be explained by its effectiveness against multiple seizure types, including generalized seizures and epileptic spasms.

In the present analysis, the prescribing profile of antiseizure medications as per the WHO prescribing indicators was evaluated. The mean number of medications prescribed per patient visit was 3. These findings agree with the research conducted by Upadhya et al, where it was revealed that the mean number of medications prescribed per patient visit was 2.61. In our study, 122 (63%) of prescriptions were written in generic form. This is required to improve rational prescribing. Our finding is the same as research done by Upadhya et al., where it was estimated that 29.83% of the medications were written by the generic nomenclature. A total of 162(82.66%) were written from the WHO model list of EDL. This finding is the same as the other studies organized by Upadhya et al., 65.47% of the total drugs were prescribed as per the WHO model list of EDL [17]. There was 0% prescription of injectable drugs, which is in concordance with other studies [18,19]. These findings indicate moderate adherence to rational prescribing principles. In comparison, Kaur et al. reported that 96.6% of drugs were prescribed from the National List of Essential Medicines, suggesting slightly better adherence to essential medicine guidelines [13]. Similar observations have been reported in earlier studies where generalized epilepsy constituted most seizure types [20,21].

In addition to antiseizure drugs, calcium and vitamin supplements were the most prescribed concomitant medications in the present study. Such supplements are frequently used in patients receiving long-term antiepileptic therapy due to concerns regarding bone health and potential nutritional deficiencies associated with certain antiseizure medications.

Overall, the findings of the present study highlight the predominant use of valproate and the frequent utilization of polytherapy in pediatric epilepsy management at the study center. These results underscore the need for continuous monitoring of prescribing patterns to ensure rational drug use and adherence to evidence-based epilepsy treatment guidelines.

Study limitations

The present study has certain limitations. Dose range, dosing frequency, weight-based dosing, adherence, and long-term outcome are important considerations in pediatric epilepsy management; these parameters were not systematically collected and analyzed in the current study.

## Conclusions

The present study provides an overview of the prescribing pattern of antiseizure medications among pediatric patients with epilepsy. Generalized seizures, particularly GTCS and epileptic spasms, were the most common seizure types observed in the study population. This study demonstrates that valproate remains the most prescribed antiseizure drug in pediatric epilepsy, possibly due to its broad-spectrum use across multiple seizure types, with polytherapy predominating (65%). While generic prescribing (63%) and EDL adherence (82.66%) show partial alignment with WHO rational prescribing standards, gaps remain. The high polytherapy rate and continued phenytoin use warrant evaluation against current ILAE guidelines. Future prospective studies should assess seizure control outcomes, adverse effects, and prescribing appropriateness to guide quality improvement initiatives.
